# Application of Fluorescence Two-Dimensional Difference In-Gel Electrophoresis as a Proteomic Biomarker Discovery Tool in Muscular Dystrophy Research

**DOI:** 10.3390/biology2041438

**Published:** 2013-12-02

**Authors:** Steven Carberry, Margit Zweyer, Dieter Swandulla, Kay Ohlendieck

**Affiliations:** 1 Department of Biology, National University of Ireland, Maynooth, Kildare, Ireland; E-Mail: steven.carberry.is@gmail.com; 2 Department of Physiology II, University of Bonn, Bonn D-53115, Germany; E-Mails: margit.zweyer@ukb.uni-bonn.de (M.Z.); dieter.swandulla@ukb.uni-bonn.de (D.S.)

**Keywords:** cvHsp, diaphragm, DIGE, Duchenne muscular dystrophy, dystrophinopathy, gel electrophoresis, HspB7, *mdx*, mouse model, parvalbumin

## Abstract

In this article, we illustrate the application of difference in-gel electrophoresis for the proteomic analysis of dystrophic skeletal muscle. The *mdx* diaphragm was used as a tissue model of dystrophinopathy. Two-dimensional gel electrophoresis is a widely employed protein separation method in proteomic investigations. Although two-dimensional gels usually underestimate the cellular presence of very high molecular mass proteins, integral membrane proteins and low copy number proteins, this method is extremely powerful in the comprehensive analysis of contractile proteins, metabolic enzymes, structural proteins and molecular chaperones. This gives rise to two-dimensional gel electrophoretic separation as the method of choice for studying contractile tissues in health and disease. For comparative studies, fluorescence difference in-gel electrophoresis has been shown to provide an excellent biomarker discovery tool. Since aged diaphragm fibres from the *mdx* mouse model of Duchenne muscular dystrophy closely resemble the human pathology, we have carried out a mass spectrometry-based comparison of the naturally aged diaphragm *versus* the senescent dystrophic diaphragm. The proteomic comparison of wild type *versus mdx* diaphragm resulted in the identification of 84 altered protein species. Novel molecular insights into dystrophic changes suggest increased cellular stress, impaired calcium buffering, cytostructural alterations and disturbances of mitochondrial metabolism in dystrophin-deficient muscle tissue.

## 1. Introduction

Skeletal muscle proteomics is concerned with the global analysis of protein populations from voluntary contractile tissues. Starting material may consist of crude extracts from total muscle preparations, protein constellations from defined muscle-associated cell types, isolated organelles, supramolecular protein complexes or the fibre secretome. The separation of muscle proteins is usually performed by gel electrophoresis and/or liquid chromatography and followed by the computer-assisted analysis of proteomic maps. In order to reproducibly generate peptide signatures of separated proteins, standardized digestion protocols are applied in muscle proteomics. Mass spectrometry is the method of choice for the rapid and reliable identification of individual protein species by their peptide fingerprint or amino acid sequence. Immunoblotting surveys, immunofluorescence microscopy, biochemical assays and functional testing are usually carried out to verify large-scale proteomic data. Comprehensive reviews have critically examined the impact of mass spectrometry-based proteomics in basic and applied myology [1,2,3]. In the long-term, proteomic biomarker discovery promises to be instrumental for the swift identification of novel indicators that may decisively increase our knowledge base of basic physiological and complex pathophysiological mechanisms, as well as improve a variety of diagnostic, prognostic and therapeutic approaches in the field of neuromuscular disorders [4].

Various forms of high-resolution two-dimensional gel electrophoresis are routinely used for separating complex protein mixtures [5,6,7]. In the case of muscle proteomics, gel electrophoresis is highly suitable for the high-throughput analysis of the most abundant muscle proteins, such as myosins, actins, troponins, tropomyosins, glycolytic enzymes, mitochondrial proteins, cytoskeletal proteins and molecular chaperones [8]. Although the exact number of proteins representing the skeletal muscle proteome is not known, the accessible portion of proteins probably make up the majority of the skeletal muscle proteome [9]. The actomyosin apparatus and its auxiliary sarcomeric elements constitute nearly half of all muscle proteins [10], the enzymes of glycolysis represent the 10 most abundant proteins of the diffusible fraction of the vertebrate muscle proteome [11], mitochondrial proteins account for approximately one fifth of the muscle protein complement [12] and chaperones are also relatively abundant in skeletal muscle tissues [13]. Thus, gel-based studies with urea-soluble proteins cover a considerable portion of the total skeletal muscle proteome and are therefore suitable for both protein cataloging exercises and comparative studies. However, certain classes of protein are clearly underrepresented in proteomic approaches that employ gel electrophoresis as its main protein separation method [8]. These types of muscle-associated proteins include especially integral membrane proteins, very high molecular mass proteins and low-abundance proteins. To overcome this technical problem, other than using sophisticated liquid chromatography approaches [14], alternative electrophoretic methods can be employed to supplement routine 2D-gel based investigations. This includes agarose 2D gel electrophoresis [15,16], Native-Blue gel electrophoresis [17,18], non-reducing/reducing diagonal 2D gel electrophoresis [19,20], off-gel electrophoresis [21,22] and on-membrane digestion of proteins separated by large one-dimensional gradient gels [23,24]. In addition to studies involving 2D gels, normal and diseased skeletal muscle tissues have been extensively studied by 1D gel electrophoresis coupled to HPLC-ESI-MS/MS analysis and quantitative approaches such as the SILAC or ICAT method [9,11,12,25,26,27]. Combined outcomes from gel-based and gel-free separation methods and data generation from labeling *versus* label-free MS analyses often result in complementary findings that can give a more comprehensive overview than that achieved by just using a single proteomic approach.

**Figure 1:**
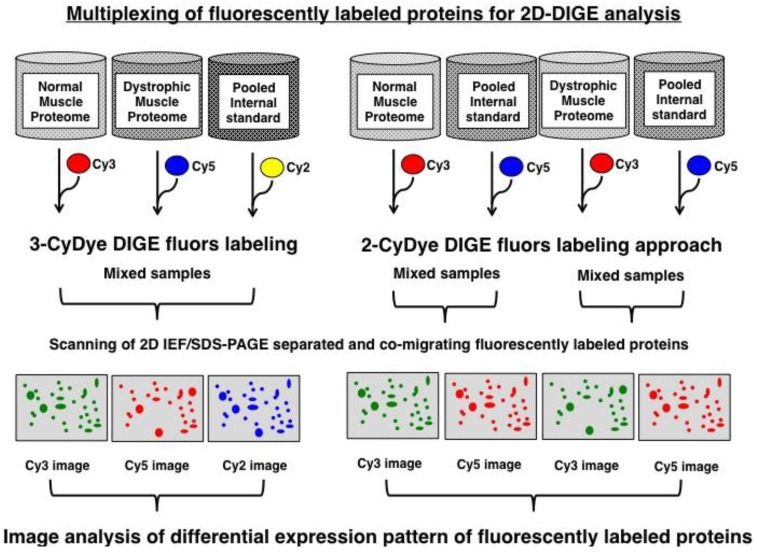
Overview of the fluorescence difference in-gel electrophoresis method: Shown are the main approaches used in fluorescence gel-based proteomics, whereby 2-dye or 3-dye difference in-gel electrophoresis (DIGE) is most commonly applied for studying global changes in complex protein populations. In the case of muscular dystrophy research, normal and dystrophic specimens are differentially labeled with CyDyes prior to electrophoresis, then proteins separated based on the unique combination of their isoeletric point and molecular masses and finally image analysis used to evaluate differential expression pattern of fluorescently labeled proteins.

Over the last few years, fluorescence two-dimensional difference in-gel electrophoresis has proven to be an excellent biomarker discovery tool for comparative studies in neuromuscular biology [28]. Originally described by Minden and co-workers [29], this key proteomic method can be used with fluorescent 2-CyDye or 3-CyDye systems to differentially label proteins from dissimilar protein mixtures prior to gel electrophoresis [30,31,32], as diagrammatically shown in Figure 1. Using optimized 2D software analysis tools [33,34], advanced DIGE analysis can highly accurately quantitate multiple protein samples on the same 2D gel, which greatly reduces the introduction of potential artifacts due to gel-to-gel variations [35,36]. For comparative muscle proteomics, routinely 50 μg protein aliquots from individual fractions are labeled with Cy2, Cy3 or Cy5 dyes. Pre-electrophoretically labeled proteins usually account for a representative proportion of key contractile, structural, metabolic and regulatory muscle proteins [37]. In general, selective labeling artifacts do not appear to play a major role in the case of soluble protein species, as suggested by the statistical analysis of the experimental variation of DIGE gels [38]. This usually eliminates the need for reverse DIGE labeling controls during routine proteomic studies. Here, we have used the 2D-DIGE technique to demonstrate its unparalleled capability as a comparative analytical tool for the characterization of animal disease models.

Animal models of human diseases are widely used in the initial identification of novel biomolecules involved in tissue degeneration [39]. In the field of experimental muscle pathology, a large number of animal genocopies are available to study the basic mechanisms of major neuromuscular diseases [40]. Although these genetic disease models are often not perfect phenocopies of a highly complex human etiology, proteomic screening of pathobiochemical changes in their expression profiles can be helpful for the initial identification of new biomarker candidates [41]. In the case of Duchenne muscular dystrophy, a lethal x-linked disorder of childhood that is caused by primary abnormalities in the *dmd* gene coding for the membrane cytoskeletal protein dystrophin [42], a variety of spontaneous and engineered animal models are available for biomedical studies [43,44]. Mammalian animal phenocopies of dystrophinopathy are highly valuable tools for elucidating the molecular pathogenesis of x-linked muscular dystrophy and developing novel therapeutic strategies [45,46]. Various muscle specimens from the *mdx* mouse model and the *grmd* dog have been used in mass spectrometry-based proteomic studies [25,26,27,37,47,48] including the successful evaluation of experimental exon-skipping therapy with novel proteomic biomarkers [49].

Building on the findings from previous proteomic studies of genetic animal models of muscular dystrophy [50] and the fact that aged muscle fibres from the dystrophic *mdx* mouse closely resemble the human pathology of Duchenne muscular dystrophy [51,52,53,54], we carried out here an optimized proteomic analysis of normal *versus mdx* tissues using aged mice. Recent proteomic investigations from our laboratories have analyzed young *mdx versus* senescent *mdx* muscles and shown a variety of protein changes in the dystrophin-deficient mouse during aging [48,55,56]. Here, we have directly compared dystrophic *mdx* diaphragm with age-matched normal muscle from senescent mice. In agreement with severe degeneration occurring in the *mdx* diaphragm, proteomics revealed considerable changes in a variety of proteins in dystrophic diaphragm tissue. Altered proteins are associated with the contractile apparatus, the extracellular matrix, the cellular stress response, metabolite transport and mitochondrial energy metabolism.

## 2. Experimental Section

### 2.1. Chemicals and Materials

For the gel electrophoresis-based proteomic analysis of aged diaphragm muscle, Coomassie Brilliant Blue, CyDye DIGE Fluor minimal dyes Cy3 and Cy5, ampholytes, acetonitrile, cover fluid and immobilised pH gradient (IPG) drystrips pH 3–11 were purchased from Amersham Biosciences/GE Healthcare (Little Chalfont, Buckinghamshire, UK). Acrylamide stock solutions were obtained as ultrapure Protogel from National Diagnostics (Atlanta, GA, USA). Laemmli-type electrophoresis buffer, protein molecular weight ladders and the Bradford reagent for protein quantification were purchased from Biorad Laboratories (Hemel-Hempstead, Hertfordshire, UK). For protein digestion, sequencing grade-modified trypsin was obtained from Promega (Madison, WI, USA). Formic acid and LC-MS Chromasolv water were from Fluka (Milwaukee, WI, USA) and spin filters were purchased from Fisher Scientific (Loughborough, UK). To verify key proteomic findings by immunoblotting, primary antibodies were obtained from Abcam, Cambridge, UK (ab111233 to the cardiovascular heat shock protein cvHSP; ab43176 to ATP synthase; ab6588 to collagen; ab28172 to prohibitin; and ab11427 to parvalbumin), Sigma Chemical Company, Dorset, UK (ab L-9393 to laminin), Santa Cruz Biotechnology, Santa Cruz, CA, USA (ab sc-33701 to β-dystroglycan), Affinity Bioreagents, Golden, CO, USA (mAb VIIID1_2_ to fast calsequestrin) and Enzo Stressgen, Victoria, BC, Canada (ab ADI-SPA-811 to heat shock protein Hsp70/72). All secondary antibodies used were obtained from Chemicon International (Temecula, CA, USA). Protease inhibitors were from Roche Diagnostics (Mannheim, Germany). Ultrapure lysine for quenching the DIGE labelling reaction and all general reagents were obtained from Sigma Chemical Company (Dorset, UK).

### 2.2. Preparation of Diaphragm Extracts from Aged mdx Mice

Since the diaphragm muscle of the aged *mdx* mouse is: (i) missing dystrophin isoform Dp427 due to a point mutation [57]; (ii) closely resembles the fibre pathology of Duchenne muscular dystrophy [58]; and (iii) exhibits an accelerated progression of muscle degeneration [51,52,53,54], we have here used 22-month old samples from *mdx* diaphragm *versus* wild type diaphragm to study global changes in dystrophinopathy. Skeletal muscle specimens were obtained from the bioresource unit of the University of Bonn [55]. Animals were kept under standard conditions and all procedures were performed in accordance with German guidelines on the use of animals for scientific experiments. Senescent *mdx* mice and age-matched non-dystrophic mice were sacrificed by cervical dislocation and post-mortem diaphragm tissues quickly removed, quick-frozen in liquid nitrogen and stored at −80 °C prior to usage. For the gel electrophoresis-based proteomic analysis of the *mdx* diaphragm, total muscle protein extracts from 4 dystrophic and 4 non-dystrophic muscle specimens were individually pulverized by grinding tissue pieces in liquid nitrogen using a mortar and pestle. A volume of 1 mL of lysis buffer, consisting of 2% IPG buffer pH 3–10, 7 M urea, 2 M thiourea, 4% CHAPS and 2% dithiothreitol, was employed to solubilize proteins from 100 mg of ground muscle powder. Importantly, the lysis buffer was supplemented with a freshly prepared protease inhibitor cocktail [37] to prevent excess protein degradation. The suspensions were centrifuged for 15 min at 15,000 × *g* at 4 °C, following gentle rocking for 60 min, and then the protein concentration determined [48].

### 2.3. Fluorescence Two-Dimensional Difference In-Gel Electrophoretic Analysis of the Diaphragm Muscle Proteome

For the pre-electrophoretic labeling of urea-soluble diaphragm muscle proteins, 50 μg protein aliquots from individual samples were fluorescently labelled with Cy3 DIGE Fluor dye [59]. The pooled internal standard was labelled with Cy5 DIGE Fluor dye. Individual samples were mixed with the appropriate amount of dye at pH 8.5, carefully vortexed and then incubated on ice for 30 min in the dark. The reaction was quenched by the addition of 10 mM lysine [60]. Following the incubation of the dye-protein mixture with lysine for 10 min on ice in the dark, fluoresecently labelled protein samples were immediately loaded onto IPG strips for electrophoretic separation in the first dimension. The gel electrophoretic separation of diaphragm proteins was performed with a total amount of 100 μg protein per analytical 2D-DIGE gel. Standardized two-dimensional gel electrophoresis was carried out with isoelectric focusing in the first dimension using 24 cm pH 3–11 strips in a IPGphor system from Amersham Biosciences/GE Healthcare (Little Chalfont, Buckinghamshire, UK) using the following running conditions: 4 h at 80 V, 2 h at 100 V, 1.5 h at 500 V, 1.5 h at 1,000 V, 1 h at 2,000 V, 1 h at 4,000 V, 2 h at 6,000 V and a final 2.5 h step at 8,000 V. IPG strips were then equilibrated twice for 20 min using 6 M urea, 30% glycerol, 2% SDS and 100 mM Tris-HCl, pH 8.8. The first incubation step was carried out in the presence of 100 mM dithiothreitol and the second incubation step using 0.25 M iodoacetamide. Subsequently IPG strips were washed in SDS running buffer (125 mM Tris, 0.96 M glycine, 0.1% (w/v) sodium dodecyl sulfate) and placed on top of 12.5% slab gels and proteins separated in the second dimension with the help of an Amersham EttanDalt-twelve system [59]. Analytical slab gels were run in parallel at 0.5 W/gel for 60 min and then 15 W/gel until the blue dye front had disappeared from the bottom of the gel. A Typhoon Trio variable mode imager was used to visualize electrophoretically separated and DIGE-labelled diaphragm proteins. Protein expression changes between *mdx* specimens and non-dystrophic specimens were analyzed using Progenesis *SameSpots* analysis software [61] (Non Linear Dynamics, Newcastle upon Tyne, UK). Individual gels were warped to a single master gel and the following parameters used during analysis: *n* = 4; *p* < 0.05; and a power value of >0.8. Diaphragm proteins with a significantly changed concentration in *mdx versus* normal specimens were picked for tryptic digestion from Coomassie Blue-stained preparative gels [59].

### 2.4. Mass Spectrometric Identification of Diaphragm Proteins

The unequivocal identification of individual diaphragm proteins, which exhibited a changed abundance in the aged *mdx* model of Duchennne muscular dystrophy, was carried out with corresponding protein spots from Coomassie-stained pick gels. Spots were excised, washed, destained and then digested with a previously optimized in-gel tryptic digestion protocol [62] and resulting peptide populations evaluated by electrospray ionization LC-MS/MS analysis [63] using a Model 6340 Ion Trap LC/MS apparatus from Agilent Technologies (Santa Clara, CA, USA). Drying of peptide mixtures was carried out by vacuum centrifugation and samples then resuspended in MS-grade distilled water and 0.1% (v/v) formic acid, spun down through spin filters and added to LC-MS vials for mass spectrometric identification. Separation of peptides was performed with a nanoflow Aligent 1200 series system. Peptide mixtures were loaded into the enrichment at a capillary flow rate set to 2 μL/min with a mix of 0.1% (v/v) formic acid and 50% (v/v) acetonitrile and formic acid at a ratio of 19:1. The voltage was set to 2,000 V. Database searches were carried out using Mascot MS/MS Ion search. Criterion for each search was set at (i) species *Mus musculus*; (ii) two missed cleavages by trypsin; (iii) variable modification: oxidation of methionine; (iv) fixed modification: carboxymethylation of cysteines and (v) mass tolerance of precursor ions ±2 Da and product ions ±1 Da. 

### 2.5. Bioinformatics Analysis of Protein Classes and Potential Protein Interactions

For the determination of clustering of molecular functions and the identification of potential protein interactions of the mass spectrometrically identified proteins with a changed abundance in dystrophic *mdx* diaphragm muscle, standard bioinformatics software was used. Analyses were performed with the PANTHER ([64]; version 8.1) comprehensive database of protein families for the cataloging of molecular functions [65] and the STRING ([66]; version 9.1) database of known and predicted protein interactions that include direct physical and indirect functional protein associations [67].

### 2.6. Immunoblot Analysis

For the independent verification of key proteomic hits, the abundance of laminin, β-dystroglycan, collagen, cvHsp, Hsp70, prohibitin, parvalbumin, calsequestrin and ATP synthase was evaluated by immunoblotting of *mdx* diaphragm samples *versus* normal muscle samples. One-dimensional gel electrophoresis and immunoblot analysis were carried out by a standard protocol, as previously described in detail [62]. The electrophoretic separation of diaphragm proteins from 22-month old *mdx* mice and age-matched normal mice was performed with 5%–10% gradient SDS-PAGE gels. Electrophoretic transfer was carried out at 100 V for 70 min using Whatman Protan nitrocellulose sheets and a Transblot Cell from BioRad Laboratories (Hemel Hempstead, Hertfordshire, UK). Blots were blocked for 1 hour with a 5% (w/v) fat-free milk protein solution in phosphate-buffered saline [68]. Following incubation with 1:1,000 diluted primary antibodies for 3 h with gentle agitation at 4 °C, membranes were washed 3 times for 10 min and then re-incubated for 1 h at 4 °C with secondary peroxidase-conjugated antibodies diluted in blocking solution. Finally, blots were washed again and the immuno-decorated protein bands visualized by the enhanced chemiluminescence method. Densitometric scanning and evaluation of immunoblots was performed with a HP PSC-2355 scanner and ImageJ (NIH, Bethesda, MD, USA) and GraphPad Prism (San Diego, CA, USA) software.

## 3. Results

### 3.1. Overview of Proteomic Surveys of the mdx Animal Model of Duchenne Muscular Dystrophy

**Figure 2:**
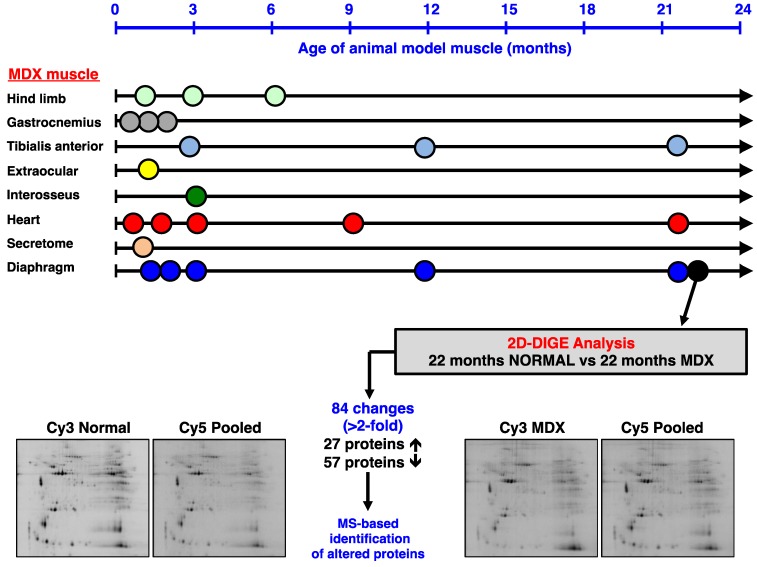
Mass spectrometry-based proteomic profiling of the *mdx* mouse model of Duchenne muscular dystrophy: The upper panel of the figure summarizes the types of muscle and age groups of *mdx* mice that have been used over the last few years to identify global changes in the dystrophic muscle proteome. All listed proteomic studies have been recently discussed in comprehensive reviews of the proteomics of the dystrophin-glycoprotein complex and dystrophinopathy [50,69]. The lower panel gives an overview of the difference in-gel electrophoretic (DIGE) analysis of the aged *mdx* diaphragm muscle presented in this report.

The optimization of animal models is a crucial part of biomedical proteomics and proteomic biomarker discovery. In the field of muscular dystrophy research, the *mdx* mouse is a widely used model system to study the molecular pathogenesis of dystrophinopathy and the suitability of novel experimental therapies to counteract dystrophic symptoms. We have recently reviewed the past usage of mass spectrometry-based proteomics to study the dystrophin-glycoprotein complex and secondary changes in the dystrophic *mdx* mouse [69]. Figure 2 summarizes the various age groups and types of muscle evaluated by proteomics and also gives an outline of the comparative proteomic approach used in this report. It is important to stress that the majority of muscles in the *mdx* mouse exhibit moderate symptoms, such as segmental necrosis in hind limb muscles. Certain *mdx* muscles, such as extraocular fibres or the *interosseus* muscle are even less necrotic than *tibialis anterior*, *extensor digitorum longus*, *gastrocnemius* or *soleus* muscles. In contrast, the *mdx* diaphragm muscle is severely affected by the deficiency in the membrane cytoskeletal protein dystrophin [58], and aging was shown to exacerbate the dystrophic phenotype [55]. Thus, the senescent *mdx* diaphragm muscle most closely resembles the human pathology. We therefore optimized here the usage of the *mdx* mouse for animal model proteomics. Using fluorescence difference in-gel electrophoresis, we have created a proteomic reference map of the senescent *mdx* diaphragm. This represents a highly improved proteomic application within the field of muscular dystrophy research.

### 3.2. Fluorescence Two-Dimensional Difference In-Gel Electrophoretic Analysis of the Naturally Aged *versus* the Aged mdx Diaphragm Muscle

**Figure 3:**
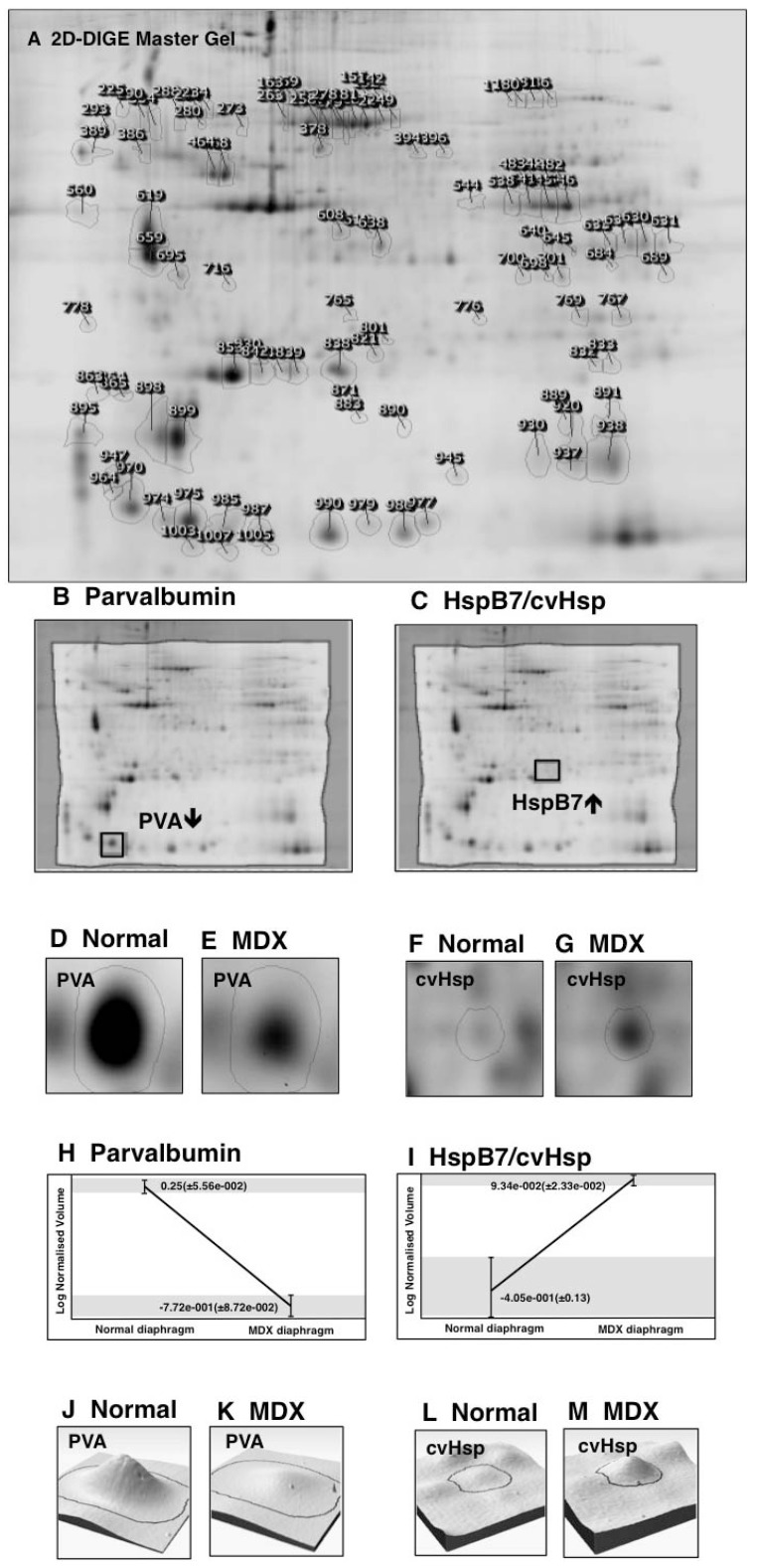
Image analysis of fluorescent DIGE gels representing normal *versus* dystrophic diaphragm muscle: Shown are a 2D-DIGE master gel used for the analysis of aged *mdx* diaphragm (**A**), the visualization of the altered expression levels of the cytosolic Ca^2+^-binding protein parvalbumin (PVA; **B**, **D**, **E**) and the small molecular chaperone cvHsp (**C**, **F**, **G**), the variability of detected alterations between normal and dystrophic samples (**H**, **I**) and the graphical presentation of the drastic decrease in parvalbumin (**J**, **K**) and the up-regulation of cvHsp (**L**, **M**) in dystrophic muscle. In this study, a Typhoon Trio variable mode imager was employed for the visualization of electrophoretically separated and CyDye-labelled diaphragm proteins. Expression changes of proteins between wild type samples and dystrophic *mdx* samples were determined with the help of Progenesis SameSpots analysis software.

**Figure 4:**
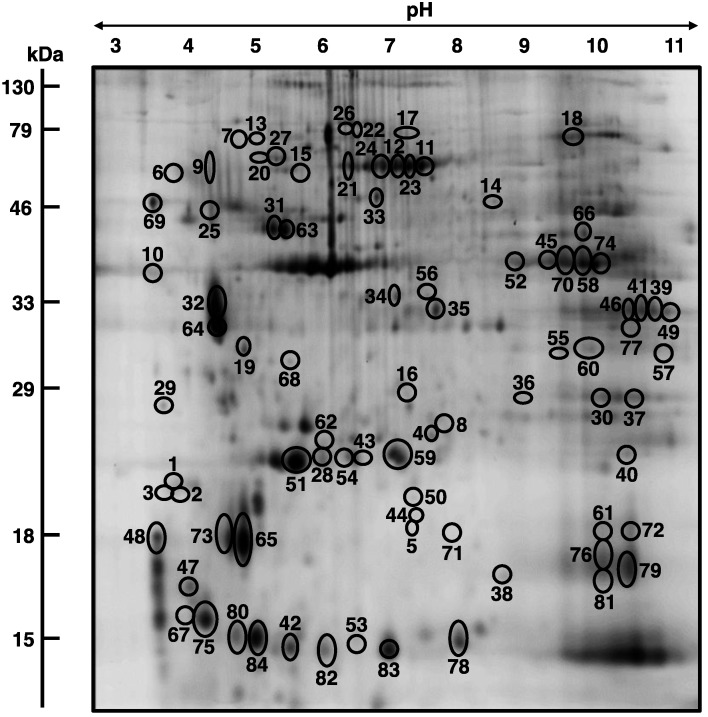
2D-DIGE master gel of the aged *mdx* diaphragm muscle: Shown is a 2D master gel that is representative of the findings of the fluorescence two-dimensional difference in-gel electrophoretic analysis of the aged *mdx* diaphragm. Protein spots with a significant change in expression levels between normal and dystrophic specimens are marked by circles and are numbered 1 to 84. See Table 1 for the mass spectrometric identification of individual diaphragm-associated proteins. The pH-values of the first dimension gel system and molecular mass standards of the second dimension are indicated on the top and on the left of the panels, respectively.

In order to determine the extent of secondary changes in the senescent *mdx* diaphragm proteome due to deficiency in the membrane cytoskeletal protein dystrophin, the urea-soluble protein repertoire from 22-month old wild type *versus* age-matched dystrophic diaphragm muscle was investigated. Following fluorescent tagging with the CyDye Cy3 of wild type or *mdx* samples, as well as fluor labeling of the pooled standard using the CyDye Cy5, a detailed densitometric analysis of two-dimensional gels was performed with a Typhoon Trio variable imager and Progenesis 2D analysis software. Figure 3 gives an overview of this standard image analysis approach used in skeletal muscle proteomics and highlights the opposite changes in the expression of the cytosolic Ca^2+^-binding protein parvalbumin *versus* the small heat shock protein HspB7/cvHsp in the dystrophic diaphragm. The proteomic comparison of wild type *versus mdx* diaphragm muscle identified 84 altered protein species in 22-month old muscle extracts. A 2D-DIGE master gel of the *mdx* diaphragm muscle is shown in Figure 4. The 2D protein spots with a significant change in abundance are marked by circles and their numbering from 1 to 84 corresponds to the list of identified proteins in Table 1.

### 3.3. Mass Spectrometric Identification of Altered Proteins in Aged mdx Diaphragm Muscle

The mass spectrometric identification of the 27 increased and 57 decreased protein species in the dystrophin-deficient and aged diaphragm is listed in Table 1, as well as their accession number, MS/MS score, isoelectric point, molecular mass, number of peptides, percent sequence coverage and fold change. Identified proteins ranged in molecular mass from 11.9 kDa (Parvalbumin) to 87.7 (Nebulin fragment), and isoelectric points ranged from p*I* 4.07 (Troponin C) to p*I* 9.19 (Nebulin fragment). Most of the identified muscle-associated proteins belong to the contractile apparatus, the cellular stress response, the extracellular matrix, ion homeostasis and energy metabolism. An increased concentration was shown for dermatopontin (spots 1 to 3), the small heat shock protein HspB7/cvHsp (spot 4), myosin light chain 6B (spot 5), unnamed protein products (spots 7, 17 and 26), beta-actin (spot 10), heat shock protein Hsp70 cognate (spots 11, 21 and 22), mitochondrial stress-70 protein (spots 12 and 24), smooth muscle gamma-actin (spot 13), a fragment of nebulin (spot 14), lumican (spot 15), prohibitin (spot 16), transferrin (spot 18), annexin A5 (spot 19), heat shock protein 8 (spot 23), Fmod protein (spot 25) and 78 kDa glucose-regulated protein (spot 27).

**Table 1:** List of identified proteins that exhibit a drastic change in abundance in the aged *mdx* diaphragm as revealed by 2D-DIGE analyses.

Spot No.	Protein Name	Accession No.	MS/MS Score	p*I*	Da	Number of peptides	Coverage (%)	Fold change
1	Dermatopontin	NP_062733	73	4.7	24,559	5	28	5.9
2	Dermatopontin	NP_062733	111	4.7	24,559	7	37	4.4
3	Dermatopontin	NP_062733	100	4.7	24,559	6	26	3.4
4	Heat shock protein HspB7 (beta7, cvHsp)	NP_038896	87	5.95	18,681	5	38	3.3
5	Myosin light chain 6B	NP_758463	181	5.41	22,851	14	74	3.1
6	Tropomyosin, beta chain isoform 1	NP_033442	319	4.66	32,933	22	61	2.9
7	Unnamed protein product	BAE35818	286	5.78	70,776	15	30	2.8
8	Myosin light chain 1/3, isoform 1f, muscle	NP_067260	97	4.98	20,697	2	12	2.7
9	Tropomyosin, beta chain isoform 1	NP_033442	107	4.66	32,933	11	35	2.7
10	Actin, beta (aa 27–375)	CAA27396	124	5.78	39,451	9	21	2.6
11	Heat shock protein Hsp70 cognate	AAA37869	110	5.37	71,025	11	23	2.6
12	Mitochondrial stress-70 protein	BAA04493	57	5.91	73,773	1	1	2.6
13	Actin, gamma, smooth muscle	AAC52237	81	5.36	43,258	5	16	2.6
14	Nebulin (fragment)	AAF59979	53	9.19	87,694	4	7	2.5
15	Lumican	AAB35361	103	5.84	38,733	2	5	2.4
16	Prohibitin	NP_032857	350	5.57	29,860	15	69	2.4
17	Unnamed protein product	BAC34145	129	5.75	70,766	8	14	2.4
18	Transferrin	AAL34533	168	6.92	78,832	19	24	2.3
19	Annexin A5	NP_033803	249	4.83	35,788	16	45	2.3
20	Alpha-fetoprotein	AAA37190	112	5.47	48,819	2	6	2.2
21	Heat shock protein Hsp70 cognate	AAA37869	110	5.37	71,025	11	23	2.2
22	Heat shock protein Hsp70 cognate	AAA37869	77	5.37	71,025	4	6	2.2
23	Heat shock protein 8	AAH66191	110	5.28	71,060	11	23	2.2
24	Mitochondrial stress-70 protein	BAA04493	71	5.91	73,773	5	9	2.1
25	Fmod protein	AAH52673	92	5.56	46,637	2	4	2.1
26	Unnamed protein product	BAC34145	254	5.75	70,766	8	17	2.1
27	78 kDa glucose-regulated protein	BAA11462	112	5.09	72,528	7	15	2.0
28	Myosin light chain 1/3, isoform 1f, muscle	NP_067260	222	4.98	20,697	15	63	−2.0
29	Parvalbumin, alpha	NP_038673	156	5.02	11,923	6	57	−2.0
30	Malate dehydrogenase	AAA39509	90	8.93	36,052	3	11	−2.1
31	ATP synthase, beta-subunit	AAB86421	98	5.14	56,344	2	4	−2.1
32	Tropomyosin, beta chain	gi|11875203	180	4.66	32,933	11	32	−2.1
33	Alpha-fetoprotein	AAA37190	95	5.47	48,819	2	5	−2.1
34	Isocitrate dehydrogenase subunit alpha	NP_083849	242	6.27	40,077	6	20	−2.1
35	L-lactate dehydrogenase B chain	NP_032518	54	5.7	36,839	5	12	−2.1
36	Actin, alpha, cardiac	gi|387090	51	5.23	42,048	2	7	−2.1
37	Coiled-coil-helix-coiled-coil-helix domain-containing protein 3, mitochondrial	NP_079612	80	8.56	26,550	5	24	−2.1
38	Cu/Zn superoxide dismutase	1513495A	147	6.03	15,926	5	41	−2.1
39	Malate dehydrogenase mitochondrial	NP_032643	256	8.93	36,053	12	45	−2.1
40	Peroxiredoxin Prdx1	NP_035164	97	8.26	22,394	11	47	−2.1
41	Malate dehydrogenase mitochondrial	NP_032643	343	8.93	36,053	15	56	−2.1
42	Cytochrome c oxidase, subunit Va	CAA34085	125	6.08	16,252	8	48	−2.2
43	ATP synthase, mitochondrial F0 complex	AAH16547	148	5.52	18,810	5	47	−2.2
44	Heat shock protein Hsp beta-6	NP_001012401	130	5.64	17,568	6	51	−2.2
45	Creatine kinase, M-type	NP_031736	276	6.58	43,250	9	27	−2.2
46	Malate dehydrogenasemitochondrial	NP_032643	243	8.93	36,053	9	36	−2.2
47	Troponin C, skeletal muscle	NP_033420	238	4.07	18,156	3	28	−2.2
48	Troponin C, skeletal muscle	NP_033420	774	4.07	18,156	8	53	−2.3
49	Malate dehydrogenase 2, mitochondrial	ABD77283	365	7.7	32,121	7	28	−2.3
50	Myoglobin	NP_038621	61	7.07	17,117	1	9	−2.3
51	Myosin light chain 1/3, isoform 1f, muscle	NP_067260	93	4.98	20,697	4	17	−2.3
52	Creatine kinase, M-type	NP_031736	297	6.58	43,250	10	28	−2.3
53	Fatty acid-binding protein FABP3	NP_034304	157	6.11	14,810	6	48	−2.3
54	Calcium-binding protein NCS-1-like	XP_003945963	48	6.49	12,957	1	17	−2.4
55	Electron transferring flavoprotein, alpha	AAH03432	449	8.62	35,366	11	44	−2.4
56	Isocitrate dehydrogenase subunit alpha	NP_083849	167	6.27	40,077	6	19	−2.4
57	Malate dehydrogenase 2, mitochondrial	ABD77283	85	7.7	32,121	2	8	−2.4
58	Creatine kinase, M-type	NP_031736	404	6.58	43,250	11	33	−2.4
59	ATP synthase, subunit d, mitochondrial	NP_082138	168	5.52	18,796	11	70	−2.4
60	Electron transfer flavoprotein, subunit alpha	NP_663590	195	8.62	35,336	8	39	−2.5
61	Cofilin-2	NP_031714	158	7.66	18,814	5	36	−2.5
62	Adenylate kinase, isoenzyme AK1	NP_067490	104	5.7	23,334	6	36	−2.5
63	ATP synthase, beta-subunit	AAB86421	301	5.14	56,344	6	16	−2.6
64	Tropomyosin, beta chain	gi|11875203	129	4.66	32,920	10	25	−2.6
65	Myosin light chain MLC2, skeletal muscle	NP_058034	93	4.82	19,059	2	8	−2.7
66	Fumarate hydratase 1	AAH06048	173	9.12	54,568	6	17	−2.8
67	Myosin light chain 1/3, isoform 1f, muscle	NP_067260	201	4.98	20,697	11	53	−2.9
68	Ubiquinone biosynthesis protein COQ9	NP_080728	150	5.6	35,235	5	21	−3.0
69	Calsequestrin, skeletal muscle	AAC63616	143	3.93	45,619	8	22	−3.1
70	Creatine kinase, M-type	NP_031736	505	6.58	43,250	10	31	−3.1
71	Myoglobin	NP_038621	124	7.07	17,117	6	48	−3.1
72	Myoglobin	NP_038621	59	7.07	17,117	1	11	−3.2
73	Myosin light chain MLC2, skeletal muscle	NP_058034	267	4.82	19,059	15	68	−3.4
74	Creatine kinase, M-type	NP_031736	266	6.58	43,250	11	31	−3.5
75	Myosin light chain 1/3, isoform 1f, muscle	NP_067260	87	4.98	20,697	2	12	−3.6
76	Myoglobin	NP_038621	91	7.07	17,117	2	21	−3.7
77	Malate dehydrogenase	AAA39509	126	8.93	36,052	5	20	−3.7
78	Fatty acid-binding protein FABP3	NP_034304	426	6.11	14,810	11	77	−3.7
79	Myoglobin	NP_038621	129	7.07	17,117	6	46	−4.0
80	Parvalbumin, alpha	NP_038673	285	5.02	11,923	6	57	−4.0
81	Myoglobin	NP_038621	89	7.07	17,117	6	41	−4.3
82	Fatty acid-binding protein FABP3	NP_034304	188	6.11	14,810	7	63	−5.3
83	Fatty acid-binding protein FABP3	NP_034304	532	6.11	14,810	9	71	−5.3
84	Parvalbumin alpha	NP_038673	431	5.02	11,923	8	57	−10.5

In contrast, a decreased concentration was established for parvalbumin (spots 29, 80 and 84), various isoforms of malate dehydrogenase (spots 30, 39, 41, 46, 49, 57 and 77), ATP synthase (spots 31, 43, 59 and 63), isocitrate dehydrogenase (spot 35), actin (spot 36), mitochondrial coiled-coil-helix-coiled-coil-helix domain-containing protein 3 (spot 37), Cu/Zn superoxide dismutase (spot 38), peroxiredoxin (spot 40), cytochrome c oxidase (spot 42), heat shock protein Hsp beta-6 (spot 44), creatine kinase (spots 45, 52 and 74), troponin C (spots 47 and 48), myoglobin (spots 50, 71, 72, 76, 79 and 81), fatty acid-binding protein FABP3 (spots 53, 78, 82 and 83), calcium-binding protein NCS-1 (spot 54), electron transferring flavoprotein (spots 55 and 60), isocitrate dehydrogenase (spot 56), cofilin (spot 61), adenylate kinase AK1 (spot 62), myosin light chain MLC2 (spots 65 and 73), fumarate hydratase (spot 66), ubiquinone biosynthesis protein COQ9 (spot 68) and calsequestrin (spot 69).

Proteins with differential expression patterns were identified as myosin light chain 1/3, alpha-fetoprotein and the beta chain of tropomyosin. Individual 2D protein spots representing the muscle isoform 1f of myosin light chain 1/3 were shown to be both increased (spot 8) and decreased (spots 28, 51, 67 and 75). In the case of the beta chain of tropomyosin, both increased (spots 6 and 9) and decreased (spots 32 and 64) concentrations of individual isoforms were determined. The alpha-fetoprotein exhibited increased (spot 20), and decreased (spot 33) levels. Although spots 12, 50, 54 and 72 were only recognized by 1 peptide, they were included in Table 1 based on their MS/MS scores of 57, 61, 48 and 59, respectively. All other proteins were identified by at least 2 peptides. 2D protein spots with a molecular mass and/or isoelectric point that differ substantially from the biochemical properties of its identified protein are probably due to streaking effects based on cross-contamination with abundant proteins, extensive post-translational modifications, proteolytic fragmentation or non-specific protein clustering.

### 3.4. Changed Protein Classes and Predicted Interaction Patterns of Altered Proteins

**Figure 5:**
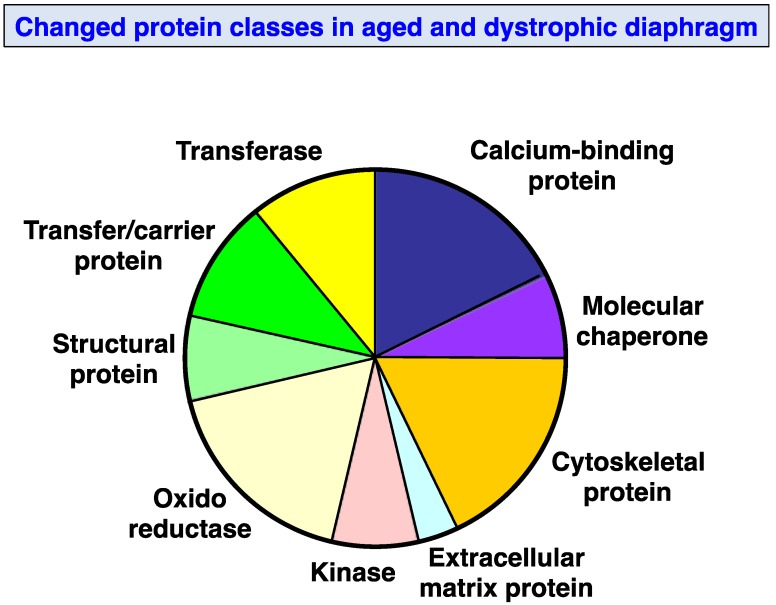
Molecular function of altered *mdx* diaphragm-associated proteins: The bioinformatics software programme PANTHER (database; version 8.1; [64,65]) was applied to identify the clustering of molecular functions of the mass spectrometrically identified proteins with a changed abundance in aged *mdx* diaphragm as compared to normal muscle (Table 1).

Figure 5 provides an overview of the distinct classes of proteins, which have been identified by proteomics to be altered in *mdx* diaphragm muscle. The application of the PANTHER database of protein families [64,65] resulted in the cataloging of molecular functions of the identified muscle proteins as being involved in ion homeostasis, the cellular stress response, cytoskeletal stabilization, extracellular matrix organization, structural integrity, metabolic transportation and metabolism. Large groups of proteins were represented by calcium-binding proteins, cytoskeletal proteins and oxidoreductases. Frequent changes were also observed in molecular chaperones, kinases, structural proteins, transferases and carrier proteins. Relatively few alterations were shown to occur in extracellular matrix proteins.

**Figure 6:**
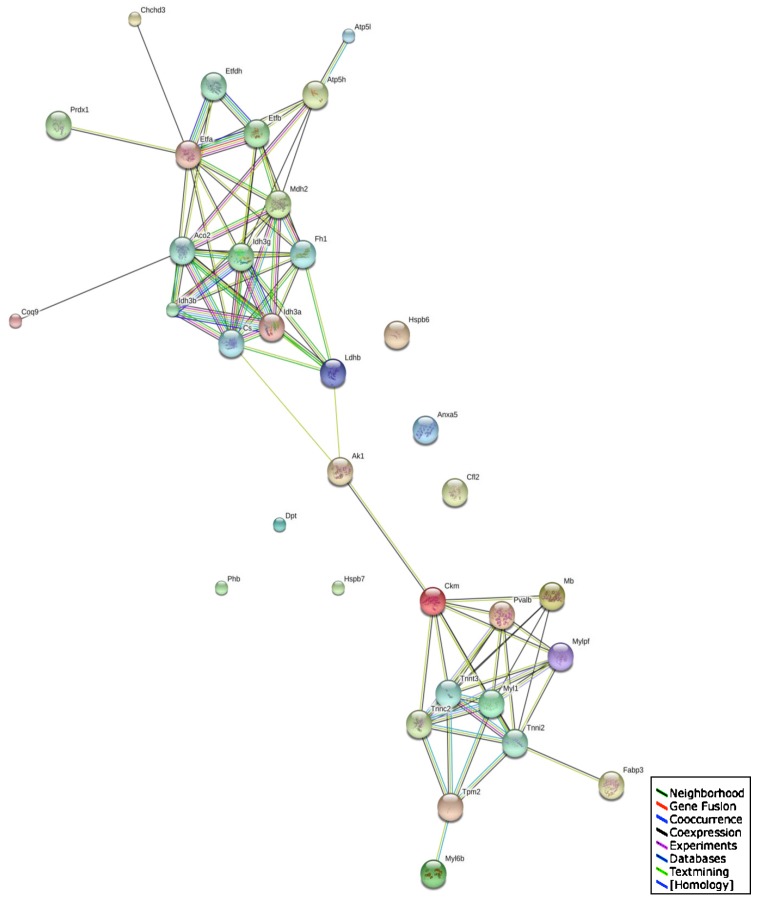
Interaction map of altered *mdx* diaphragm-associated proteins: The bioinformatics STRING database (version 9.1; [66,67]) was used to generate a protein interaction map with known and predicted protein associations that include direct physical and indirect functional protein linkages of the mass spectrometrically identified proteins with a changed abundance in aged *mdx* diaphragm as compared to normal muscle (Table 1).

The STRING database of direct physical and indirect functional protein interactions [66,67] was employed to evaluate potential protein networks within the proteomic data set generated by this study of the aged *mdx* diaphragm. This bioinformatic analysis revealed a relatively complex interaction map (Figure 6). Two large clusters were shown to exist that are linked via adenylate kinase and creatine kinase interactions. Since both proteins were demonstrated to be reduced in dystrophin-deficient fibres, this indicates that more severe downstream effects on various proteins may occur within these apparent protein clusters. One of the identified protein networks consists especially of enzymes involved in mitochondrial metabolism, such as ATP synthase, isocitrate dehydrogenase, aconitase and malate dehydrogenase. Another protein cluster contains several major contractile elements, including tropomyosin, troponin and myosins. Important ion handling proteins and metabolite transporters are also proposed to interact with these protein complexes.

### 3.5. Immunoblot Analysis of Altered Proteins in Aged mdx Diaphragm Muscle

**Figure 7:**
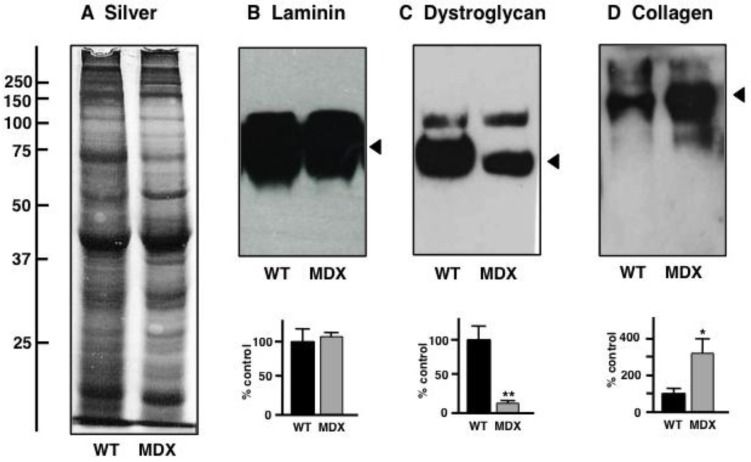
Gel electrophoretic and immunoblot analysis of the aged *mdx* diaphragm muscle: Shown is a silver-stained gel (**A**) and representative immunoblots (**B**–**D**) with expanded views of antibody-decorated bands. Lanes 1 and 2 represent preparations from non-dystrophic wild type (WT) *versus* dystrophic (MDX) diaphragm muscle, respectively. Immuno-decoration was carried out with primary antibodies to laminin (**B**), the dystrophin-associated glycoprotein β-dystroglycan (**C**) and collagen (**D**). Below the individual immunoblots are shown panels, which give a graphical representation of the immuno-decoration levels in normal *versus mdx* preparations (Student’s *t*-test, unpaired; *n* = 4; * *p* < 0.05; ** *p* < 0.01).

In order to independently verify the status of potential biomarker candidates with a drastically altered expression in aged *mdx* diaphragm as revealed by proteomics, comparative immunoblotting was employed. Figure 7A shows a silver-stained 1D gel of non-dystrophic wild type *versus* dystrophic *mdx* diaphragm preparations. The gel exhibits relatively comparable overall protein banding patterns in both types of muscle. Immunoblotting of laminin revealed no significant difference in expression levels of this component of the basal lamina (Figure 7B). To confirm the mutant status of the *mdx* diaphragm specimens used in this study, the concentration of β-dystroglycan was evaluated. Immunoblotting clearly showed a drastic reduction in this dystrophin-associated glycoprotein (Figure 7C). In contrast, the extracellular matrix protein collagen was found to be significantly increased in its expression, which agrees with the senescent and dystrophic status of the aged *mdx* diaphragm muscle.

**Figure 8:**
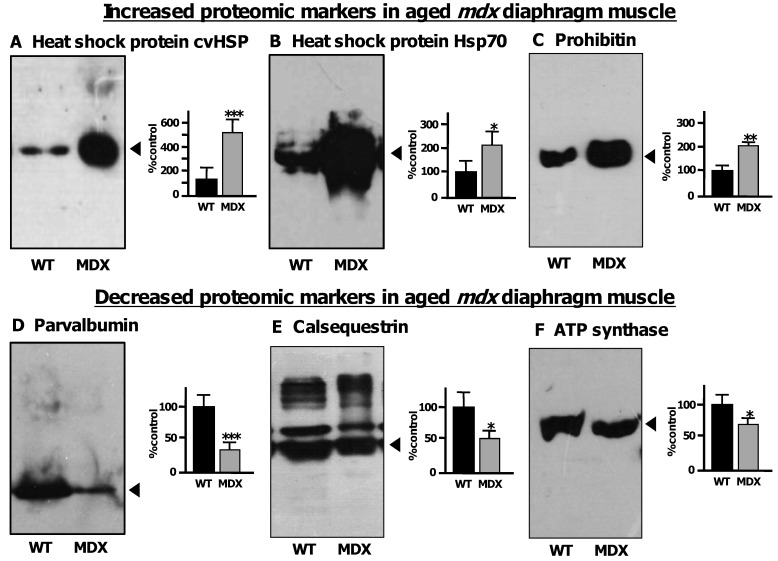
Immunoblot analysis of the aged *mdx* diaphragm muscle: Shown are representative immunoblots with expanded views of antibody-decorated bands. Lanes 1 and 2 represent preparations from non-dystrophic wild type (WT) *versus* dystrophic (MDX) diaphragm muscle, respectively. Immuno-decoration was carried out with primary antibodies to heat shock protein cvHsp (**A**), heat shock protein Hsp70 (**B**), prohibitin (**C**), parvalbumin (**D**), the luminal Ca^2+^-binding protein calsequestrin (**E**) and the mitochondrial enzyme ATP synthase (**F**). Besides the individual immunoblots are shown panels, which give a graphical representation of the immuno-decoration levels in normal *versus mdx* preparations (Student’s *t*-test, unpaired; *n* = 4; * *p* < 0.05; ** *p* < 0.01, *** *p* < 0.001).

Figure 8 shows immunoblot labeling that clearly verified the increase of 3 distinct diaphragm proteins and the concomitant decrease of 3 other diaphragm proteins. An increased concentration in dystrophic muscle was shown for the small heat shock protein cvHsp, the molecular chaperone Hsp70 and the mitochondrial protein prohibitin (Figure 8A–C). In contrast, the cytosolic Ca^2+^-binding protein parvalbumin, the luminal Ca^2+^-binding protein calsequestrin and the mitochondrial enzyme ATP synthase were confirmed to be decreased in senescent *mdx* diaphragm (Figure 8D–F). The immunoblot analysis of calsequestrin with monoclonal antibody VIIID1_2_ to the fast CSQ isoform showed besides the major 60 kDa band of its monomer also several high molecular mass bands that represent previously characterized calsequestrin-like proteins [70].

## 4. Discussion and Conclusions

The comprehensive cataloguing of tissue-specific mRNA profiles, protein populations and metabolite fluxes in normal biosystems is now a major undertaking in the modern biosciences following the successful determination of key mammalian genomes. In addition to the establishment of biomolecular constellations under normal physiological conditions, the high-throughput screening of disease-related alterations in the genome, transcriptome, peptidome, proteome and metabolome promises the systematic identification of novel biomarkers of pathophysiological adaptations and degenerative pathways. Importantly, the differential large-scale analysis of the full complement of genes, mRNAs, peptides, proteins and metabolites present in a defined biological entity, such as body fluids, subcellular fractions, cells, tissues or organisms, is an unbiased technology-driven approach [71,72,73]. Thus, key protein biochemical techniques such as mass spectrometry-based proteomics represent a hypothesis-generating method in discovery mode that aims at the swift establishment of biomarker signatures. In contrast to conventional hypothesis-driven bioresearch that routinely focuses on a select number of protein factors, disease proteomics attempts to evaluate global changes in the entire protein collection of a cellular system [74]. Besides using secreted or leaked proteins as a rich source for biomarker discovery [75], changes in the abundance, oligomerisation, interactions and/or post-translational modifications in tissue-associated proteins are an excellent basis for establishing novel candidate markers [76].

Since single gene disorders that result from a distinct genetic rearrangement or mutations in a unique gene, such as Duchenne muscular dystrophy, have a defined primary genetic marker, these inherited diseases usually exhibit a corresponding and diagnostically accessible faulty protein as an expression marker [42]. This makes this particular type of genetic disorders relatively easy to evaluate on the molecular genetic level, as compared to many complex disorders with potential interplays between genetic susceptibility, life style and environmental factors. However, single gene disorders of the neuromuscular system often show highly complex secondary alterations in their proteome, making it difficult to understand potential hierarchies in degenerative pathways and thus hamper the development of reliable stage-specific diagnostic or prognostic procedures. In this respect, muscle proteomics suggests itself as a highly appropriate screening technique for identifying novel indicators of disease progression [4]. The comparative analysis of the naturally aged diaphragm *versus* the dystrophic *mdx* diaphragm presented here illustrates that the fluorescence difference in-gel electrophoresis method, in combination with optimized animal model proteomics, can be extremely helpful for the establishment of new protein markers involved in the molecular pathogenesis of dystrophinopathy. Biomarker discovery studies with rodent models are relatively cost-effective and aging studies can be carried out in a convenient period of time. In addition, more complex animal models of muscular dystrophy have been employed in the search for new markers of dystrophinopathy. This includes the *grmd* dog model of Duchenne muscular dystrophy [25].

**Figure 9:**
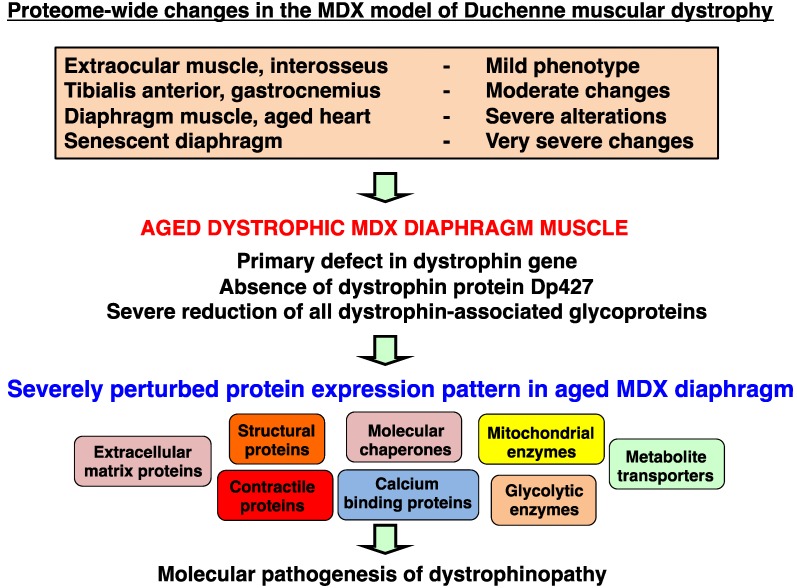
Proteomic profile of the aged *mdx* diaphragm muscle: The diagram summarizes the main classes of diaphragm proteins identified by the proteomic profiling of the aged and dystrophin-deficient *mdx* mouse model of X-linked muscular dystrophy. The severely perturbed protein expression pattern of dystrophic muscle tissue includes extracellular matrix proteins, contractile proteins, structural proteins, molecular chaperones, mitochondrial enzymes, glycolytic enzymes, calcium-binding proteins and metabolite transporters. Thus, the deficiency in dystrophin isoform Dp427 and concomitant reduction in dystrophin-associated glycoproteins in the dystrophic sarcolemma appears to trigger a large range of secondary abnormalities in muscular dystrophy.

The diagrammatic presentation in Figure 9 summarizes the proteomic findings from the analysis of the senescent *mdx* mouse model of Duchenne muscular dystrophy. Global alterations in contractile proteins, structural proteins, extracellular proteins, calcium-binding proteins, molecular chaperones, mitochondrial enzymes, glycolytic enzymes and metabolite transporters indicate rearrangements within the actomyosin apparatus, changes in the cytoskeletal network and its indirect linkage to the extracellular matrix, impaired ion handling, an enhanced cellular stress response and considerable metabolic disturbances. Overall, these results imply that protein expression patterns are drastically perturbed in the Dp427-deficient *mdx* diaphragm and that these pathobiochemical changes are more intense as compared to *mdx* hind limb muscle [26,47,56]. High levels of dermatopontin and a concomitant increase in collagen, as previously shown by proteomics [55], agree with a progressive accumulation of connective tissue in the dystrophic diaphragm [77]. The degeneration and/or re-modeling of the contractile apparatus, as shown here to include troponins, tropomyosin, actin and myosin light chains, and elevated levels of fibrosis appear to be accompanied by drastic changes in various heat shock proteins. This includes the up-regulation of the muscle-specific molecular chaperone cvHsp and several isoforms of Hsp70, which agrees with the general idea of severe cellular stress in dystrophinopathy [37]. Interestingly, increased levels of a nebulin fragment were observed indicating a potential compensatory mechanism to stabilize the weakened sarcomeric structure by strengthening the nebulin-associated thin filament in dystrophic fibres [10]. The 2-fold increase in transferrin could be due to changes in iron absorption and usage in muscular dystrophy. Since circulating transferrin delivers essential iron to tissue proteins [78], the elevated concentration of this iron transporter indicates disturbed iron homeostasis in degenerating *mdx* diaphragm muscle.

Previous studies have shown that skeletal muscles from the *mdx* mouse exhibit impaired mitochondrial metabolism resulting in decreased intramuscular ATP levels. Mitochondrial abnormalities include the pathophysiological uncoupling of oxidative phosphorylation and a concomitant reduced capacity for maximal ATP synthesis [79] and a disruption of the subsarcolemmal localization of mitochondria that promotes metabolic inefficiency and thus restricts the maximal generation of ATP [80]. In analogy to the findings from a recent proteomic study of the aged *mdx* heart [48], the senescent *mdx* diaphragm seems to also show a bioenergetic dysfunction of mitochondria and this suggests that aged mitochondria are unable to meet ATP demand in dystrophic muscle fibres. A decreased concentration was observed for key mitochondrial enzymes, including isocitrate dehydrogenase, malate dehydrogenase, cytochrome c oxidase, electron transferring flavoprotein and ATP synthase. In addition, the proteomic findings of a reduced concentration of the essential and rate-limiting metabolite transporters for the utilization of fatty acids and oxygen, fatty acid binging protein FABP3 and the intracellular oxygen transporter myoglobin, agree with the concept of impaired oxidative metabolism.

Over the last few years, comparative studies with *mdx* muscle tissue extracts have indicated significant changes in the enzyme adenylate kinase AK1 [37,47,81], the Ca^2+^-binding protein calsequestrin and its high-molecular-mass isoforms [68,82], the cytosolic Ca^2+^-binding protein parvalbumin [83], the actin binding protein profilin [26], the oxygen carrier myoglobin [81], the molecular chaperone cvHsp/HspB7 [37,49], different isoforms of annexin [26], the lysosomal-associated membrane protein LAMP1 [27], the enzyme carbonic anhydrase CA3 [56], the fatty acid binding protein FABP3 [26], the mitochondrial enzyme isocitrate dehydrogenase [26,83,84], the extracellular matrix protein dermatopontin [55] and the ion transporter transferrin [48,85]. These proteomic screening studies of the *mdx* mouse were carried out with a great variety of subtypes of muscle, various tissue extracts or subcellular fractions, diverse protein separation approaches, differing labeling methods and numerous mass spectrometric techniques [26,27,37,47,48,49,55,56,81,82,83,84,85].

The proteomic analysis presented here has confirmed that the concentration of cvHsp, dermatopontin, annexin, profilin and transferrin is drastically increased and that the abundance of calsequestrin, parvalbumin, myoglobin, isocitrate dehydrogenase, and adenylate kinase is severely reduced in the aged *mdx* diaphragm model of Duchenne muscular dystrophy. This makes these proteins excellent biomarker candidates of dystrophinopathy, which might be helpful in improving diagnostic, prognostic or therapeutic approaches. In conclusion, the proteomic analysis shown here establishes the suitability of the fluorescence difference in-gel electrophoresis method for studying highly complex contractile tissues in a comparative fashion and demonstrates that the aged *mdx* diaphragm represents a suitable model system to study secondary effects of dystrophin deficiency in skeletal muscle tissue. 
